# Immune Modulation in HLA-G Expressing Head and Neck Squamous Cell Carcinoma in Relation to Human Papilloma Virus Positivity: A Study From Northeast India

**DOI:** 10.3389/fonc.2019.00058

**Published:** 2019-02-25

**Authors:** Neelanjana Sarmah, Munindra Narayan Baruah, Shashi Baruah

**Affiliations:** ^1^Immunology and Immunogenetics Laboratory, Department of Molecular Biology and Biotechnology, Tezpur University, Tezpur, India; ^2^North East Cancer Hospital and Research Institute, Guwahati, India

**Keywords:** HLA-G, carcinogenesis, HPV–human papilloma virus, HNSCC (head and neck squamous cell carcinoma), immune tolerance, SOCS, immunomodulator, immune surveillance

## Abstract

**Background:** Tumor specific ectopic expression of the immunomodulatory molecule, HLA-G is known to mediate immune tolerance and promote carcinogenesis. Viruses too employ strategies to evade immune surveillance. Considering the role of both HLA-G and HPV in tumor growth and progression, it is pertinent to investigate the relationship between HLA-G and HPV in context of immune modulation in HNSCC.

**Method:** A hospital based case–control study was conducted in histopathologically confirmed HNSCC tissues. HLA-G isoform expression and HPV association studies were carried out and mRNA levels of HLA-G, markers of proliferation and differentiation (ki-67, keratin 18, cyclin D1), immune checkpoint molecules (IL-10, PD-1. TGF-β), SOCS (SOCS1 and SOCS3) and pro-inflammatory cytokine IFN-γ were determined.

**Results:** Higher expression of HLA-G was noted in HPV positive tumors (5.14 fold, *p* = 0.002). HLA-G7 was the most frequent isoform (29/80) found in HNSCC particularly in HPV positive tumors (13/16). In HPV negative tumors, all the checkpoint molecules were upregulated along with pro–inflammatory IFN-γ. In contrast, in HPV positive tumors, IFN-γ expression was higher (2.12 fold) but levels of IL-10, PD-1, TGF-β, SOCS1 and SOCS3 were markedly lower (fold change of IL-10 = 0.37, PD1 = 0.41, TGF-β = 0.17, SOCS1 = 0.055, SOCS3 = 0.027). HPV positive tumors were more proliferative and differentiated with higher expression of ki-67 and keratin18 (6.25 fold, *p* = 0.079 and 10.62 fold, *p* = 0.009). Decreased expression of cyclin D1 was noted in HPV positive tumors (6.94 fold, *p* = 0.006) than HPV negative tumors (17.69 fold). Also, HLA-G7 expressing HPV positive tumors showed lowest expression of cyclin D1. Interestingly, SOCS showed normal expression in HLA-G7 expressing HPV negative tumors (1.2 and 1.4 fold). IFN-γ was downregulated in HPV positive tumors without HLA-G7 (0.31 fold).

**Conclusion:** Our data suggests that SOCS were downregulated irrespective of HLA-G positivity and IFN- γ expression appeared to be mediated by HLA-G. SOCS are reported to have anti-tumor activity and also SOCS and soluble HLA-G are known to interfere with cell cycle progression. Hence, through regulating HLA-G expression, HPV positive tumors could mediate immune suppression by manipulating SOCS, IFN-γ, IL-10 and cyclin D1 pathways which needs further exploration.

## Introduction

Head and Neck Squamous Cell Carcinoma (HNSCC) is considered the sixth most common cancer with an annual incidence of approximately 400,000 worldwide (1). In Northeast (NE) India, it acquires 30–40% of cancers at all sites and is the sixth most common cause of death in males and seventh in females (2). Prolonged exposure to alcohol, tobacco, and Human Papilloma Virus (HPV) infections are known risk factors of HNSCC (3).

There is a two-way dynamic interaction between cancer cells and the host immune system that leads to the dictation of the pace of tumor growth. Various strategies have been developed and applied by tumor cells to avoid recognition and destruction by different immune effectors (4, 5) and escape immune surveillance. One of the strategies is induced aberrant expression of the non-classical class I molecule Human Leukocyte Antigen-G (HLA-G) (6). Tumor-mediated ectopic expression of HLA-G suppresses the function of T cells and natural killer (NK) cells and induces expansion of an immunosuppressive T cell subset (7). The alternative splicing of the HLA-G primary transcript can generate seven different isoforms, four being membrane-bound (HLA-G1, HLA-G2, HLA-G3, HLA-G4) and three being soluble (HLA-G5, HLA-G6, HLA-G7). Most polymorphic sites of HLA-G are present at 5′URR (upstream regulatory region) which affects the transcription of the HLA-G gene and 3′UTR (untranslated region) which influences mRNA processing and stability (8).

HPVs are double-stranded DNA viruses with a genome comprised of six “early” genes (E1–E2, E4–E7) and two “late” genes (L1 and L2) (9). E5, E6, and E7 are the three major viral oncoproteins responsible for altering cell cycle regulation and blocking tumor suppressor pathways and reported to contribute to tumerogenesis (10). The most common HPV types associated with cancer are HPV 16 and 18. HPV positive HNSCC tumors show different patient and clinical characteristics besides etiological and epidemiological differences (11).

Several studies have been reported to determine the association of HLA-G polymorphisms with the HPV infection (12, 13). In a recent genome-wide association study (GWAS), it was shown that oropharyngeal cancer associations were limited to the human leukocyte antigen (HLA) region and this association was considerably stronger in HPV positive cancers which suggested a correlation between HLA expression and virus-related cancers (14). Further, Lajoie et al. explained that HLA-G re-expression has been suggested as a viral escape mechanism from immunosurveillance (15). Recently, Louvanto et al. reported that HLA-G molecules played a role in the prediction of the likelihood of the newborn's for oral HPV infection at birth (16).

There are a few studies on the association between HLA-G polymorphisms with HPV infections (14–16). However, the interaction between HPV infection and HLA-G isoforms in tumors is relatively less explored. Accordingly the present study was designed to understand the interaction between HPV and HLA-G in context of immunoregulation in HNSCC tumors.

## Materials and Methods

### Study Site, Study Design, and Participants

The study was a hospital based case-control study and 85 histopathologically confirmed cases of HNSCC were included for the conduct of the study. Oncogenecity was determined by immunohistochemistry for p^16^ protein (Supplementary Figures 1A–C). The study was approved by the Institutional Ethics Committee of North East Cancer Hospital and Research Institute (NECHRI) vide sanction No-IEC/2017/03/002. The patients were informed about the study and information sheet was provided prior to collection of the specimens. Biopsy and postoperative tissue samples of approximately upto 5 cm of diameter were collected from NECHRI after obtaining a written informed consent. For staging and diagnosis of HNSCC tumors, the American Joint Committee on Cancer's TNM staging protocol was used as a reference. Histopathologically confirmed adjacent normal tissue was used as the control for performing the study.

### Extraction of DNA, RNA, and Quality Assessment of Samples

Tissue samples were collected in RNA later solution (Ambion, United States) and homogenized using a hand-held tissue grinder (G-Biosciences, United States). DNA and RNA were extracted using Allprep DNA/RNA/protein kit (Qiagen, Hilden, Germany) according to the manufacturer's instructions. RNA was reverse transcribed to cDNA using High capacity cDNA reverse transcription kit (Invitrogen, Applied Biosystems, Foster City, United States) according to the manufacturer's instructions. The quantity and quality of the isolated genomic DNA and cDNA were confirmed by Nano-Vue^TM^ plus spectrophotometer (GE Healthcare, Little Chalfont, United Kingdom) and agarose gel electrophoresis respectively. To assess the quality of extracted DNA and cDNA, PCR was carried out for the human β-globin gene using standard PCO3 and PCO4 oligonucleotides as described previously (17). Five samples that were negative for human ß-globulin gene amplification were excluded from the study and all other 80 samples were subjected to HLA-G isoform expression, HPV association, and gene expression studies.

### HLA-G Isoform Expression Study

PCR reaction was standardized for exon 2, exon 3 and exon 4 of as described by Hviid et al. (18) in a Veriti PCR (Applied Biosystems, United States) to amplify the exonic regions of HLA-G gene using cDNA as the template. After optimization of PCR conditions, expression study of HLA-G isoforms was carried out in 80 HNSCC tissue samples and their adjacent normal tissues in a total volume of 15 μL using 2 μL of template cDNA, 1X PCR buffer, 1.5 mM MgCl_2_, 200 μM of each deoxynucleotide triphosphate (dATP, dTTP, dCTP, dGTP), 0.5 μM of each primers and 1.5 U of Taq DNA polymerase. To determine whether the isoforms are membrane bound or soluble, PCR was performed using exon 5 specific primer sequences as described by Van and Ober (19). Isoforms of HLA-G were classified according to the presence the exon 2, exon 3, exon 4 and exon 5 as described by Amiot and Samson (20). Specificity of band amplification was determined by sequencing of 15% of the amplicons.

### HPV Association Study

The HPV association study was standardized by PCR using HPV 18 positive control obtained from the National Institute of Biomedical Genomics (NIBMG, Kalyani, West-Bengal, India). PCR reaction was carried out for the L1 gene of outer region of HPV plasmid by using sequence-specific primers for 80 DNA samples obtained from tumor tissue in a total volume of 15 μL using 2 μL of template DNA, 1X PCR buffer, 1.5 mM MgCl2, 200 μM of each deoxynucleotide triphosphate (dATP, dTTP, dCTP, dGTP), 0.5 μM of each primers and 1.5 U of Taq DNA polymerase. Thermal cycling was performed in a Veriti PCR (Applied Biosystems, USA) as described by Fontaine et al. (21). All the HPV positive samples were further gel extracted using the Qiaquick Gel extraction kit (Qiagen, Hilden, Germany) according to the manufacturer's instructions and specificity of band amplification was determined by sequencing.

### Gene Expression Analysis

Gene expression analysis was performed using SYBR green based assays (Applied Biosystems, USA) for HLA-G, interferon-γ (IFN-γ), markers of proliferation and differentiation- ki-67, keratin 18 and cyclin D1, immune checkpoint molecules- interleukin 10 (IL-10), tumor growth factor-β (TGF-β), programmed cell death protein 1 (PD1), suppressor of cytokine signaling 1 and 3 (SOCS1 and SOCS3) on StepOne Plus Real-Time PCR System (Applied Biosystems, United States). Glyceraldehyde 3-phosphate dehydrogenase (GAPDH) was used as the endogenous control for normalization of expression levels. Histopathologically confirmed adjacent normal tissues were used as the calibrators for mRNA quantification by comparative CT method. Primers for IL-10 and TGF-β were designed using Primer Express software while for other genes RT^2^ qPCR Primer Assays (Qiagen, Hilden, Germany) were used as shown in the Supplementary Table 1. Real-time PCR was carried out in 10 μL of reaction volume consisting of 1X SYBR green PCR master mix, 0.4 μM of each primer, 200 ng of cDNA and water to adjust the volume. The relative expression of the target genes was determined using the formula 2^−Δ*Δct*^ method.

### Statistical Analysis

Participants with incomplete data or incomplete investigations were excluded from analysis. Statistical analysis of the data was performed using XLSTAT 2015 version. Correlation analysis was performed between the expression of marker genes, cytokines and HLA-G using the Pearson's Correlation test. Factor analysis was carried out to determine the predictors of disease. Relationships between gene expression and clinical data were analyzed by logistic regression models. Student's *t*-test was used for comparison between the mean values. A *p* < 0.05 was considered as statistically significant.

## Results

Our study cohort comprised of individuals from three linguistic affinities namely Indo-European (IE), Austro-Asiatic (AA), and Tibeto-Burman (TB) populations in the age group of 29–84 years with a median age of 58 years. HNSCC patients were mainly from IE population (56.25%) and 63.75% of the patients were in the age group of 45- 64 years. It may be noted that in earlier studies, HNSCC was found to be associated with older age groups but in our study, we found an association of younger as well as middle-aged group (<45 = 11.25% and 45–64 = 63.75%) with the disease. 50% of the patients were in stage II of the disease and in 56.25% of the patients, adjacent nodes were involved as shown in Table 1.

**Table 1:** Clinical and demographic profile of HNSCC patients.

**Characteristic**	**Total Cases (*n* = 80)**
**GENDER**	
Male	58 (72.5%)
Female	22 (27.5%)
**AGE GROUP (YEAR)**	
<45	9 (11.25%)
45–64	51 (63.75%)
65–84	20 (25%)
**ETHNICITY**	
IE	45 (56.25%)
AA	8 (10%)
TB	27 (33.75%)
**STAGE OF TUMOR**	
Stage I	5 (6.25%)
Stage II	40 (50%)
Stage III	16 (20%)
Stage IV	19 (23.75%)
**NODE STATUS**	
No node	35 (43.75)
Node involvement	45 (56.25)
**METASTASIS**	
No metastasis	57 (71.25%)
Metastasis	3 (3.75%)
Not determined	20 (25%)

^*^ IE, Indo-European; AA, Austro-Asiatic; TB, Tibeto-Burman.

### Expression of Different Isoforms of HLA-G in Tumor Tissue

**Figure 1:**
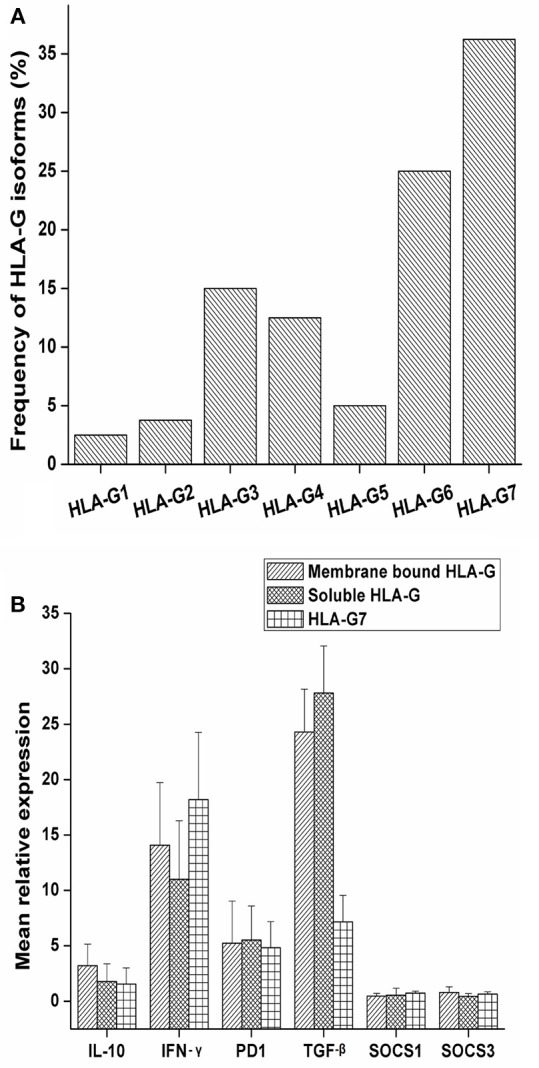
Frequency of expression of HLA-G isoforms and mRNA expression levels of IL-10, PD1, TGF-β, IFN-γ SOCS1 and SOCS3 with HLA-G isoform status. **(A)** The study was carried out in a total of 80 tumor tissue samples and frequency of isoforms in tumor tissue was determined. Overall soluble isoforms were found to be predominant (66.25%) over membrane bound isoforms (33.75%). **(B)** Samples were stratified as membrane bound HLA-G positive, soluble HLA-G positive and HLA-G7 positive. Mean relative expression was calculated by 2^∧^−ΔΔct method. Histopathologically confirmed adjacent normal tissue was used as the calibrator. Error bars in the graph represented standard deviation from the mean. IL-10 (3.20 fold), and IFN-γ (14.07 fold) expressions were higher in membrane bound isoforms as compared to soluble ones (1.77 and 11.01 fold, respectively). In contrast, soluble isoforms of HLA-G showed higher expression of TGF-β (27.08 fold). PD1 expression was found to be approximately 5 fold in both the isoforms. Considering HLA-G7 as the most frequent isoform of HLA-G expressed in tumor tissue, the samples were stratified on the basis of HLA-G7 positivity and found that expression of IFN-γ was highest in HLA-G7 expressing tumors (18.20 fold) of HNSCC.

Soluble isoforms of HLA-G (G5, G6, and G7) were predominant as compared to membrane-bound isoforms (G1, G2, G3, and G4) as given in Figure 1A. HLA-G7 was found to be the most frequent isoform with the frequency of 36.25% and HLA-G1, the full-length membrane-bound form of HLA-G was the least abundant isoform (2.5%) as given in Figure 1A. Notably, HLA-G was expressed in all HNSCC tumor tissues. HLA-G expression was also checked in adjacent normal tissues and only 20% of the tissues showed expression of HLA-G which indicates tumor-restricted expression of HLA-G in HNSCC.

### Cytokine Profile of Tumors in Relation to HLA-G Isoforms

We observed upregulations of immune checkpoint molecules IL-10, PD1, TGF-β and inflammatory molecule IFN-γ in tumor tissue while SOCS1 and SOCS3 expressions were found to be comparable to controls (Figure 1B). Comparing the expression pattern with respect to membrane-bound and soluble isoforms of HLA-G in tumor tissue, we found that IL-10 and IFN-γ expressions were higher in membrane-bound isoforms. In contrast, soluble isoforms of HLA-G showed higher expression of TGF-β as shown in Figure 1B. Considering HLA-G7 as the most frequent isoform of HLA-G expressed in tumor tissue, we stratified the samples on the basis of HLA-G7 positivity and found that expression of IFN-γ was highest in HLA-G7 expressing tumors of HNSCC. Hence, it would be interesting to ask if IFN-γ expression is due to HLA-G mediated signaling.

### HPV Status of HNSCC Tumors

A total of 16 patients were found to be HPV positive (20%) of the 80 HNSCC patients. Male individuals were found to be predominantly associated with HPV. Fifty percent of all HPV positive tumors were seen in Tibeto-Burman ethnic group. In the context of the site of the tumor, apart from the oropharynx, sinus and the oral cavity comprising of the tongue, lip, mucosa, and glottis were also seen to be associated with HPV. Oral cavity was seen to be predominantly associated with HPV infection (50%) while the frequency of association of pharynx and tonsil was found to be equal at 25% each. HPV positive tumors were found to be most frequently associated with stage II and stage III and 12/16 were primary tumors without the involvement of metastasis as given in Table 2.

**Table 2:** Clinical and demographic profile of HNSCC patients with respect to HPV positivity.

**Characteristic**	**HPV positive (*n* = 16)**	**HPV negative (*n* = 64)**
**GENDER**		
Male	12 (75%)	46 (71.87%)
Female	4 (25%)	18 (28.12%)
**AGE GROUP (YEAR)**		
<45	2 (12.5%)	7 (10.93%)
45–64	12 (75%)	39 (60.93%)
65–84	2 (12.5%)	18 (28.12%)
**ETHNICITY**		
IE	4 (25%)	41 (64.06%)
AA	4 (25%)	4 (6.25%)
TB	8 (50%)	19 (29.68%)
**SITE OF CANCER**		
Pharynx	4 (25%)	12 (18.75%)
Sinus	4 (25%)	6 (9.37%)
Oral cavity	8 (50%)	46 (71.87%)
**STAGE OF TUMOR**		
Stage I	1 (6.25%)	4 (6.25%)
Stage II	10 (62.5%)	30 (46.87%)
Stage III	4 (25%)	12 (18.75%)
Stage IV	1 (6.25%)	18 (28.12%)
**NODE STATUS**		
No node	9 (56.25%)	26 (40.62%)
Node involvement	7 (43.75%)	38 (59.37%)
**METASTASIS**		
No metastasis	12 (75%)	45 (70.31%%)
Metastasis	0 (0%)	3 (4.68%%)
Not determined	4 (25%)	16 (25%)

^*^IE, Indo-European; AA, Austro-Asiatic; TB, Tibeto-Burman.

### Proliferation and Differentiation of Tumors With Respect to HPV Positivity

**Figure 2:**
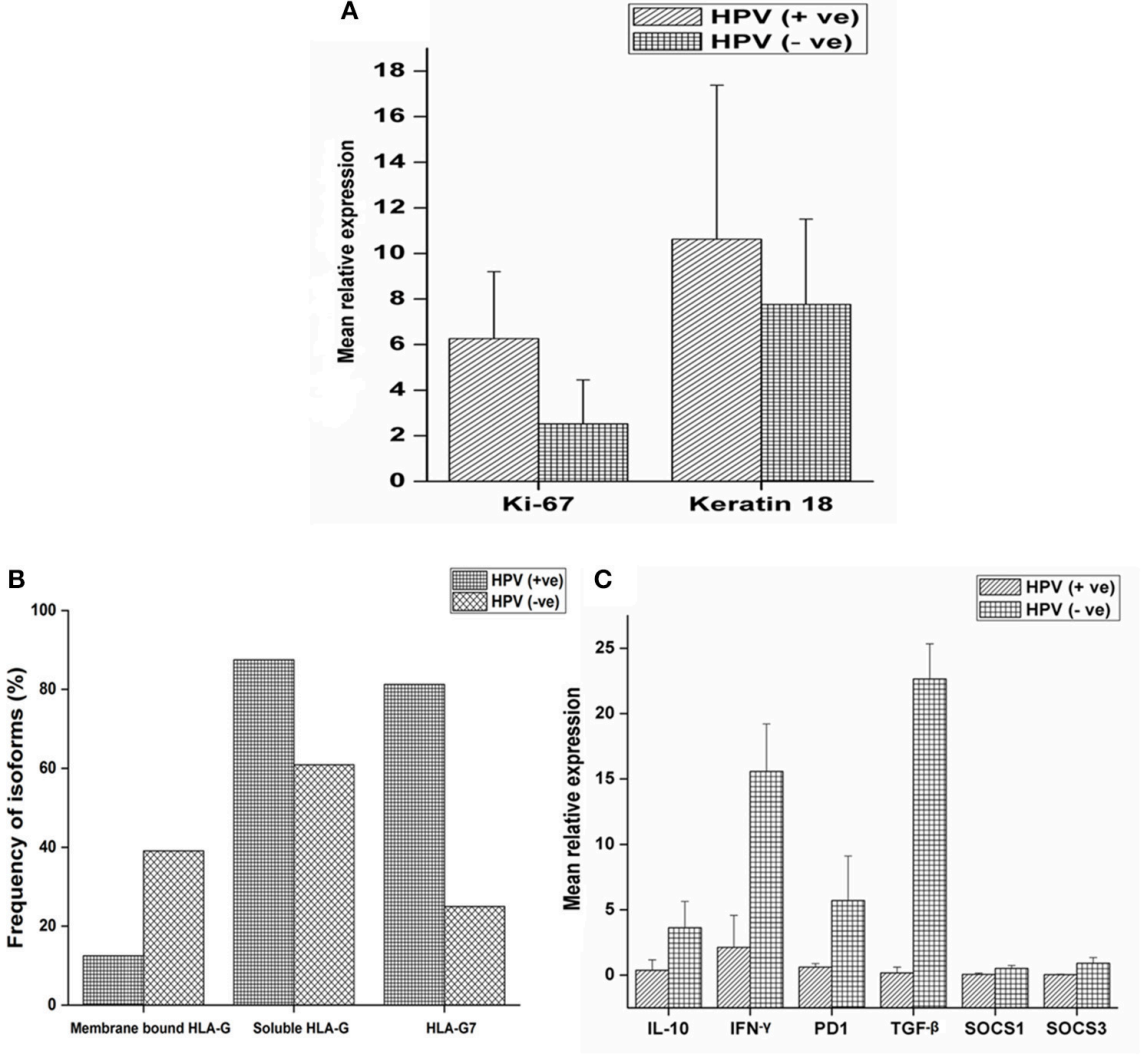
mRNA expression profile of target genes and expression of HLA-G isoforms with respect to HPV positivity. **(A)** mRNA expression profile of ki-67 and keratin18 were determined in 16 HPV positive and 64 HPV negative samples and mean relative expression was calculated by 2^∧^−ΔΔct method. Histopathologically confirmed adjacent normal tissue was used as the calibrator. Error bars in the graph represented standard deviation from the mean. Student's *t*-test was used to compare the means and *p* < 0.05 was considered as statistically significant. ki-67 and keratin 18 expressions were higher in HPV positive tumors (6.25 and 10.62 fold, respectively) compared to HPV negative tumors (2.51 and 7.76 fold, respectively). **(B)** Samples were selected and stratified as-membrane bound HLA-G with HPV, membrane bound HLA-G without HPV, soluble HLA-G with HPV, soluble HLA-G without HPV, HLA-G7 with HPV and HLA-G7 without HPV. Frequency of association of isoforms with HPV positivity was calculated. **(C)** mRNA expression profile of IL-10, TGF-β, IFN-γ, PD1, SOCS1, and SOCS3 were determined in 16 HPV positive samples and 64 HPV negative samples. Error bars in the graph represented standard deviation from the mean. p<0.05 was considered as statistically significant. TGF-β (22.66 fold), IL-10 (3.62 fold) and PD1 (5.70 fold) together with inflammatory cytokine IFN-γ (15.58 fold) upregulated in HPV negative tumors. SOCS levels were not downregulated but comparable to controls (SOCS1 = 0.50 fold and SOCS3 = 0.89 fold). But in HPV positive tumors, TGF-β (0.17 fold), IL-10 (0.37 fold) and PD1 (0.41 fold) were downregulated except inflammatory cytokine IFN-γ (2.12 fold). SOCS levels were found to be severely downregulated (SOCS1 = 0.05 fold and SOCS3 = 0.02 fold).

**Figure 3:**
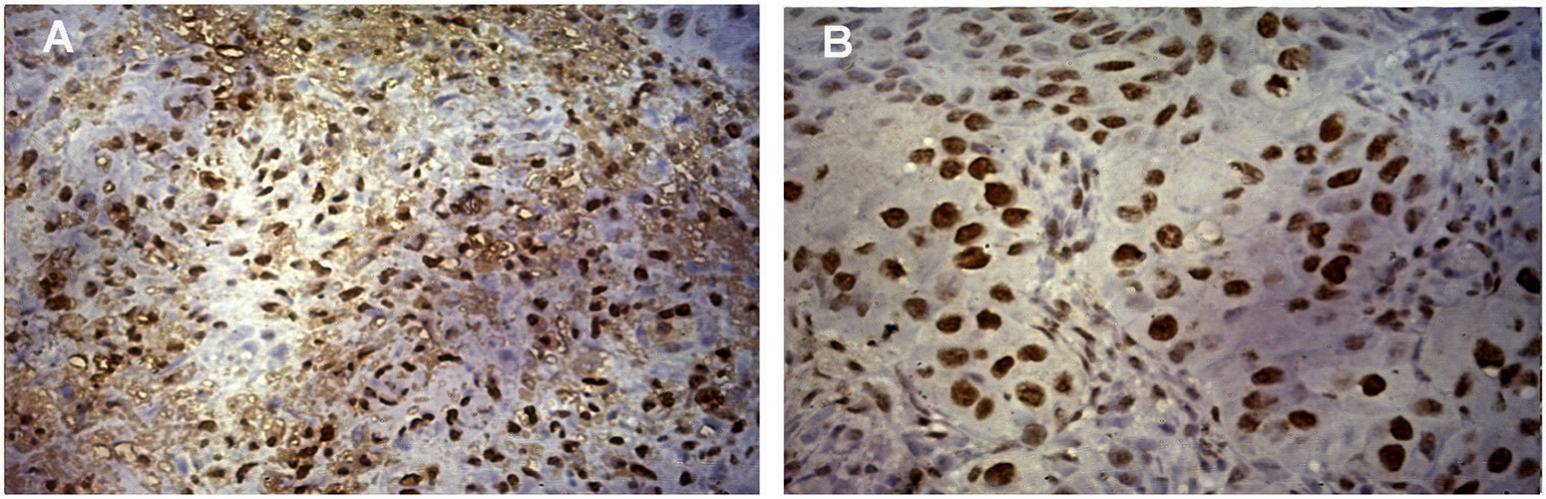
Expression of ki-67 protein in HNSCC tumor tissue. **(A)** Immunohistochemical staining of ki-67 showing the positivity of ki-67 in HPV positive HNSCC tumor cells counter stained by hematoxylin. Image was captured in Axio Vert.A1 inverted microscope (Carl Zeiss, Oberkochen, Germany) at 40X maginification. **(B)** Immunohistochemical staining of ki-67 showing the positivity of ki-67 in HPV negative HNSCC tumor cells counter stained by hematoxylin. Image was captured in Axio Vert.A1 inverted microscope (Carl Zeiss, Oberkochen, Germany) at 40X maginification.

Expression of ki-67, a marker of proliferation was higher in HPV positive tumors (Student's *t*-test, *p* = 0.079) as given in Figure 2A. ki-67 protein expression was determined by immunohistochemistry (IHC) on formalin-fixed, paraffin-embedded (FEPE) tumor tissues using monoclonal ki-67 primary antibody raised in mouse (Sigma-aldrich, Merck, Germany). Ki-67 expression was calculated according to the staining intensity and graded (from 1+ to 4+). Tumor tissues having >50% staining were considered as positive for ki-67 (Figures 3A,B). Further, logistic regression analysis taking ki-67 as the response variable revealed that of odds of overexpression of ki-67 (1.5 fold or a higher expression of ki-67) increased with HPV positivity (Odds ratio = 3.599, 95% *CI* = 0.841–15.407). Higher expression of a marker of differentiation, keratin 18 was seen in HPV positive cases (Student's *t*-test *p* = 0.009). Expression levels of ki-67 and keratin 18 were positively correlated in HPV positive tumors (Pearson *r* = 0.512 and *p* = 0.043). The proliferation of tumor cells is frequently seen with dedifferentiation of cells but, in our case, we found differentiation in tumors along with ongoing proliferation which is interesting.

### HLA-G and Its Isoform With HPV Status

HLA-G was found to be upregulated in tumor tissue. However, mean relative expression of HLA-G gene was higher in HPV positive tumors (Student's *t*-test *p* = 0.002) compared to HPV negative tumors as shown in the Supplementary Figure 2. Soluble isoforms (87.5%) were seen more frequently in HPV positive tumors than membrane-bound isoforms (12.5%) with HLA-G7 (81.25%) as the most frequent soluble isoform (Figure 2B).

### HPV Associated Cytokine Profile of Tumors

Higher expressions of immune checkpoint molecules TGF-β (*p* = 0.006), IL-10 (*p* = 0.005) and PD1 (*p* = 0.137) together with inflammatory cytokine IFN-γ (*p* = 0.031) were seen in HPV negative tumors. Weak positive correlations between of IL-10 expression with IFN-γ (Pearson *r* = 0.478 and *p* = 0.006), PD1 (Pearson *r* = 0.437 and *p* = 0.012) and TGF-β (Pearson *r* = 0.365 and *p* = 0.040) expressions were observed in HPV negative tumors suggesting the classical trend with active feedback inhibition loop. We also observed a positive correlation between expressions of IL-10 and HLA-G (Pearson *r* = 0.580 and *p* = 0.001) in case of HPV negative tumors but not in HPV positive tumors. In contrast, in HPV positive tumors, downregulation of IL-10, TGF-β and PD1 were observed but IFN-γ was upregulated (Figure 2C) which is unexpected. Notably, SOCS1 and SOCS3 expressions were severely downregulated (SOCS1, *p* = 0.053, and SOCS3, *p* = 0.050) in HPV positive tumors suggesting manipulation of SOCS expression in HPV positive tumors while in HPV negative tumors the expression was comparable to the calibrator as shown in Figure 2C.

### HPV and HLA-G7 Mediated Cytokine Environment in HNSCC Tumors

**Figure 4:**
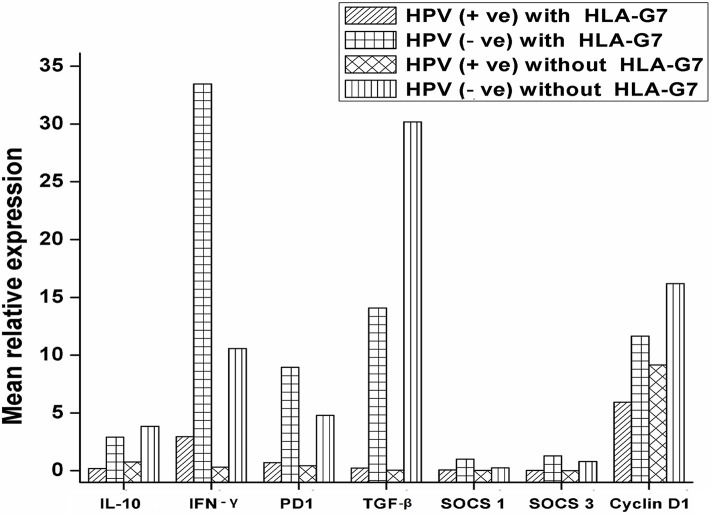
mRNA expression profile of immune regulatory molecules with HPV and HLA-G7 status. Samples were stratified with respect to HPV and HLA-G7 positivity and mean relative expressions of the target genes were compared. Data showed that when HPV was present with HLA-G7, all the immune regulatory molecules (IL-10 = 0.17 fold, PD1 = 0.49 fold, TGF-β = 0.23 fold, SOCS1 = 0.06 fold and SOCS3 = 0.03 fold) were downregulated whereas, inflammatory IFN-γ (2.97 fold) was overexpressed. In contrast, HPV negative with HLA-G7 samples showed upregulations of all the genes (IL-10 = 2.97 fold, PD1 = 8.95 fold, TGF-β = 14.07 fold, IFN-γ = 33.46 fold, SOCS1 = 1.40 fold and SOCS3 = 1.27 fold). However, HLA-G7 negative HPV positive samples showed downregulations of IFN-γ = 0.31 fold, PD1 = 0.43 fold, TGF-β = 0.05 fold, SOCS1 = 0.020 fold and SOCS3 = 0.005 fold while IL-10 level was comparable to healthy control (0.75 fold). In HLA-G7 and HPV negative samples, increased expressions of all the molecules were observed (IL-10 = 3.85 fold, PD1 = 4.79 fold, TGF-β = 30.18 fold, IFN-γ = 10.58 fold, respectively) except SOCS1 (0.25 fold) and SOCS3 (0.79 fold). Cyclin D1 was upregulated in all the cases but HPV positive HLA-G7 expressing tumors showed lowest expression of cyclin D1 (5.90 fold) as compared to HPV and HLA-G7 negative tumors (16.18 fold).

HPV and HLA-G have been reported to modulate immune responses by altering cytokine profiles and manipulating the signaling pathways in cancer. We were interested to see how the interaction between HPV and HLA-G changes the expression pattern of the immunoregulatory genes. We compared the expression profiles of regulatory molecules (IL-10, PD1, TGF-β, IFN-γ SOCS1, and SOCS3) with HPV and HLA-G7 status as HLAG-7 was the most frequent isoform found in HPV positive tumors. Our data revealed that when HPV was present with HLA-G7, all the immune regulatory molecules (IL-10, PD1, TGF-β, SOCS1, and SOCS3) were downregulated whereas, inflammatory IFN-γ was overexpressed. In contrast, HPV without HLA-G7 was contributed to downregulation of IFN-γ. When HLA-G7 expression was seen with HPV negativity, increased expression of all the molecules was observed in the tumor. This data suggested that HPV was involved in downregulation of immune checkpoint molecules while the presence of HLA-G7 leads to increased expression of IFN-γ as shown in Figure 4. Cyclin D1 was upregulated in tumors. However, expression cyclinD1 was lower (Student's *t*-test, *p* = 0.006) in HPV positive tumors compared to HPV negative cases as shown in the Supplementary Figure 2. We also checked the expression of cyclin D1 with respect to HPV positivity and with HLA-G7 status and found that expression of cyclinD1was inversely proportional to HPV positivity and HLA-G status (Figure 4).

## Discussion

HLA-G is a potent immune-modulator, which by mediating secretion of cytokines like IFN-γ is known to support cross-talk between uterine decidual cells and trophoblasts and simultaneously suppressing Natural Killer Cells and T Cell-mediated cytolysis (7) to support implantation (22). It is therefore not surprising that the ectopic expression of HLA-G is tumor tissue limited as described by Zheng et al. (23). In our study too, the expression of HLA-G was largely specific to tumor tissues and there was a predominance of soluble isoforms, particularly HLA-G7. Earlier studies have reported the association of soluble isoforms with other carcinomas including Oral Squamous Cell Carcinoma (24–28), however, to the best of our knowledge, this is the first report of the association of HLA-G7 with HNSCC tumors. Notably, we found higher expression of HLA-G in HPV positive tumors hinting at possible exploitation of HLA-G by HPV for immune manipulation. HLA-G7 was the most frequently seen isoform (13/16) in HPV positive tumors.

Our data on the expression of cytokines and immune checkpoint molecules revealed a distinct difference in the regulation of cytokine expression between HPV positive and negative tumors. In HPV negative tumors, with upregulation of IFN-γ and IL-10, checkpoint molecules were also upregulated which suggested that immune suppression in tumor microenvironment was mediated by IL-10 dependent pathways. In contrast, in HPV positive tumors, IL-10 and checkpoint molecules were downregulated but IFN-γ was upregulated. The question then arises as to how immune modulation was achieved in HPV positive tumors. To examine this further we compared the expression levels of SOCS1 and SOCS3 genes in HPV positive and HPV negative tumors. Expressions of SOCS1 and SOCS3 were markedly lower in HPV positive tumors. However, in HPV negative tumors, levels of SOCS were comparable to healthy controls. Further, HPV negative but HLA-G7 positive samples showed normal expression of SOCS indicating the role of HPV in dysregulation of SOCS. Comparable SOCS levels were also observed in HPV negative tumors without HLA-G7 which is acceptable as downregulation of SOCS in the tumor has been reported earlier (36). Studies have shown that SOCS1 has antitumor activity by preventing chronic inflammation (29, 30) and by mediating phosphorylation of the tumor suppressor p53 protein (31) which probably explains the downregulated status of SOCS1 in HPV positive tumors. Similarly, SOCS3 downregulation allows for persistence of STAT3 mediated signaling that is proposed to contribute to carcinogenesis by inducing multiple tumor-promoting genes (32). Our contention that SOCS downregulation was HPV mediated is supported by the study conducted by Ogata et al. where they too observed lower levels of SOCS in Hepatitis C Virus-infected cancerous lesions (33).

Notably, HPV tumors were seen to be more aggressive with increased levels of ki-67 (6.25 fold) than HPV negative tumors (2.51 fold). Decreased expression of cyclin D1 was noted in HPV positive tumors. Further, the highest expression of cyclinD1 was observed in HPV and HLA-G7 negative tumors and lowest was seen in HPV and HLA-G7 positive samples. These findings are in agreement with Ketroussi et al. and Bahri et al. who reported that soluble HLA-G can inhibit cell cycle progression by regulating cyclin expression (34, 35). SOCS is also known to regulate cell cycle progression via downregulating expression of cyclinD1 (36). Hence, regulation of expression of SOCS and soluble HLA-G can be considered as one of the immune escape strategies used by the HPV positive tumors to interfere with cell cycle progression and to mediate immune subversion.

Downregulation of SOCS in HPV positive tumors may be explained by lower levels of IL-10 in these tissues, which is known to induce expression of SOCS for homeostasis (37). Alternatively, it is tempting to speculate the role of HPV triggered miRNAs or promoter hypermethylation to silence SOCS (36, 38, 39). Viruses are also known to employ miRNA based strategies to manipulate the host immune responses and this needs to be explored further (40).

In HPV and HLA-G7 positive tumors, IFN-γ the pro-inflammatory cytokine was upregulated. We had only three samples that were HPV positive but HLA-G7 negative, and in these samples, IFN-γ was downregulated. Considering the small number of HPV positive HLA-G7 negative samples in our study, we are constrained to draw any conclusion but it would appear that HPV harnessed HLA-G7 modulated pathways to drive inflammatory mechanism for its growth.

## Concluding Remarks

The study evidenced increased ectopic expression of HLA-G with HPV positivity and this is the first report of expression of HLA-G7, a soluble isoform of HLA-G in HNSCC and with HPV positivity. Our findings hint that HPV positive tumors manipulate the expression of SOCS, IFN-γ, IL-10 and, cyclinD1 in partnership with HLA-G for immune evasion and to support the growth of HNSCC tumors. In conclusion, HPV harnessed HLA-G7 modulated pathways to drive inflammatory mechanism for its growth and regulate the expression of SOCS to mediate immune subversion.

## Author Contributions

NS was involved in the design of the experiments, data acquisition, and analysis, manuscript writing. MNB was involved in the study as the Clinical Collaborator and contributed to the clinical component of the manuscript. SB was involved in study design, data analysis and interpretation, formulation and critical review of the manuscript.

### Conflict of Interest Statement

The authors declare that the research was conducted in the absence of any commercial or financial relationships that could be construed as a potential conflict of interest.

## Supplementary Material

Supplementary Figure 1 Expression of p ^16^ in HNSCC tissues and normal tissue. **(A,B)** Immunohistochemical staining of p^16^ protein showing the positivity of p^16^ in HNSCC tumor tissues counter stained by hematoxylin. Image was captured in Axio Vert.A1 inverted microscope (Carl Zeiss, Oberkochen, Germany) at 40X maginification. **(C)** Immunohistochemical staining of p^16^ showing the negativity of p^16^ in normal tissue counter stained by hematoxylin. Image was captured in Axio Vert.A1 inverted microscope (Carl Zeiss, Oberkochen, Germany) at 40X maginification.

Supplementary Figure 2 mRNA expression profile of HLA-G and cyclin D1 with HPV positivity. All the 16 HPV positive samples along with 64 HPV negative samples were considered for the study and mean relative expression was calculated by 2^∧^−ΔΔct method. Histopathologically confirmed adjacent normal tissue was used as the calibrator. Error bars in the graph represented standard deviation from the mean. Student's *t* test was used to compare the means and *p* < 0.05 was considered as statistically significant. HLA-G expression was higher in HPV positive tumor (5.14 fold) compared to HPV negative tumors (3.71 fold). Alternatively, decreased cyclin D1 expression was found in HPV positive tumors (6.94 fold) than HPV negative tumors (17.69 fold).

Supplementary Table 1 Primers used for Real time PCR based gene expression study.
