# Supplementary figures and images for: Immune Modulation in HLA-G Expressing Head and Neck Squamous Cell Carcinoma in Relation to Human Papilloma Virus Positivity: A Study From Northeast India
Source: Front Oncol. 2019 Feb 25;9:58. doi: 10.3389/fonc.2019.00058 (PMC6397850; doi:10.3389/fonc.2019.00058)

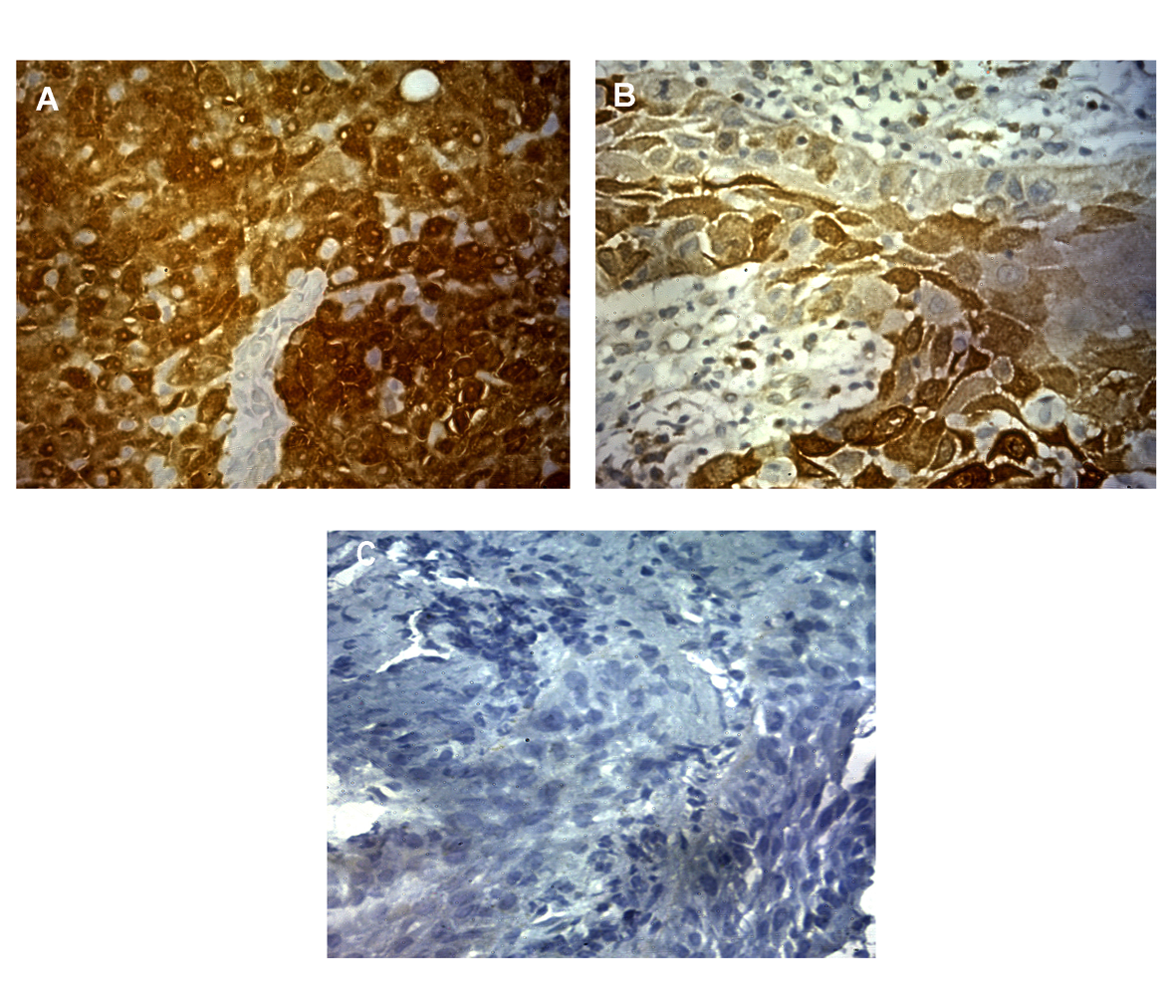

Supplement: Supplementary Figure 1 — Expression of p 16 in HNSCC tissues and normal tissue. (A,B) Immunohistochemical staining of p16 protein showing the positivity of p16 in HNSCC tumor tissues counter stained by hematoxylin. Image was captured in Axio Vert.A1 inverted microscope (Carl Zeiss, Oberkochen, Germany) at 40X maginification. (C) Immunohistochemical staining of p16 showing the negativity of p16 in normal tissue counter stained by hematoxylin. Image was captured in Axio Vert.A1 inverted microscope (Carl Zeiss, Oberkochen, Germany) at 40X maginification. [file Image_1.tif]

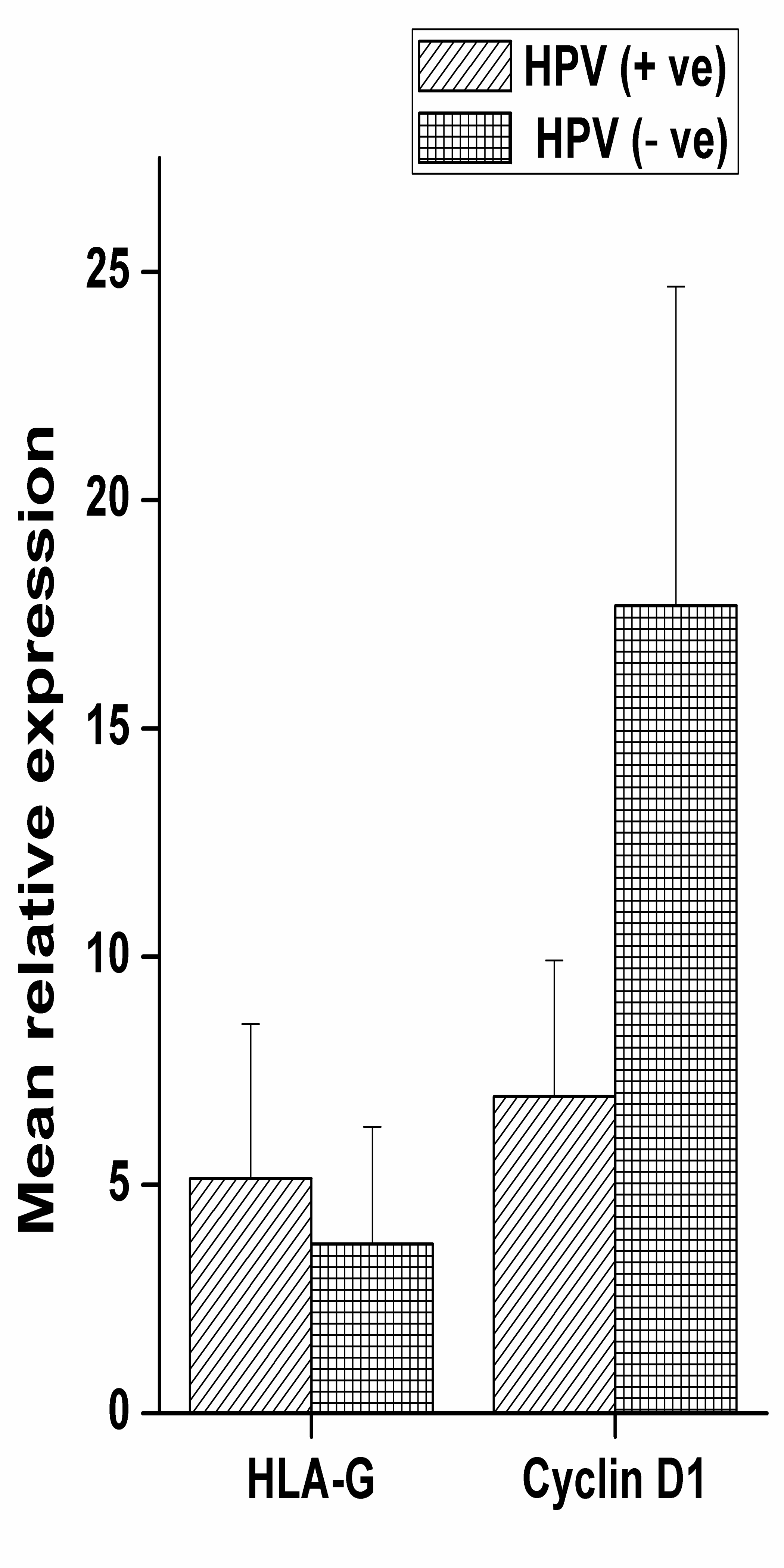

Supplement: Supplementary Figure 2 — mRNA expression profile of HLA-G and cyclin D1 with HPV positivity. All the 16 HPV positive samples along with 64 HPV negative samples were considered for the study and mean relative expression was calculated by 2∧−ΔΔct method. Histopathologically confirmed adjacent normal tissue was used as the calibrator. Error bars in the graph represented standard deviation from the mean. Student's t test was used to compare the means and p < 0.05 was considered as statistically significant. HLA-G expression was higher in HPV positive tumor (5.14 fold) compared to HPV negative tumors (3.71 fold). Alternatively, decreased cyclin D1 expression was found in HPV positive tumors (6.94 fold) than HPV negative tumors (17.69 fold). [file Image_2.TIF]
