# Supplementary material for: Immune Modulation in HLA-G Expressing Head and Neck Squamous Cell Carcinoma in Relation to Human Papilloma Virus Positivity: A Study From Northeast India
Source: Front Oncol. 2019 Feb 25;9:58. doi: 10.3389/fonc.2019.00058 (PMC6397850; doi:10.3389/fonc.2019.00058)
Supplement: Supplementary Table 1 — Primers used for Real time PCR based gene expression study. [file Table_1.docx]

**Supplementary table:** Primers used for Real time PCR based gene expression study

| **S. No** | **Gene** | **Primer** |
| --- | --- | --- |
| 1 | HLA-G | PPH23053B, RT^2^ qPCR Primer assays (Qiagen, Hilden, Germany) |
| 2 | Ki-67 | PPH01024E, RT^2^ qPCR Primer assays (Qiagen, Hilden, Germany) |
| 3 | Keratin 18 | PPH00452F,  RT^2^ qPCR Primer assays (Qiagen, Hilden, Germany) |
| 4 | Cyclin D1 | PPH00128F, RT^2^ qPCR Primer assays (Qiagen, Hilden, Germany) |
| 5 | IL-10 | FORWARD - GTGATGCCCCAAGCTGAGA  REVERSE- CCCCCAGGGAGTTCACATG |
| 6 | TGF-β | FORWARD- CTATTCAAGACCACCCACCTTCTG  REVERSE- CTCCCGGCAAAAGGTAGGA |
| 7 | IFN-γ | PPH00380C, RT^2^ qPCR Primer assays (Qiagen, Hilden, Germany) |
| 8 | PD1 | PPH13086G, RT^2^ qPCR Primer assays (Qiagen, Hilden, Germany) |
| 9 | SOCS3 | PPH00763A, RT^2^ qPCR Primer assays (Qiagen, Hilden, Germany) |
| 10 | SOCS1 | PPH00769C, RT^2^ qPCR Primer assays (Qiagen, Hilden, Germany) |
| 11 | GAPDH | PPH00150F, RT^2^ qPCR Primer assays (Qiagen, Hilden, Germany) |
